# An indigenous inland genotype of the black yeast *Hortaea werneckii* inhabiting the great pyramid of Giza, Egypt

**DOI:** 10.3389/fmicb.2022.997495

**Published:** 2022-09-26

**Authors:** Samah Mohamed Rizk, Mahmoud Magdy

**Affiliations:** Genetics Department, Faculty of Agriculture, Ain Shams University, Cairo, Egypt

**Keywords:** cultural heritage, hyper-arid environment, extremophilic fungi, biodeterioration, arid and semi-arid climate

## Abstract

Within the context of cultural heritage conservation, the biological study of tangible archeological sites is an important task to extend their existence and strengthen the transmission of their cultural value to future generations. In Egypt, a hyper-arid region, a microcolonial fungus with inky black growth was observed on a stone surface in the royal corridor of the Great Pyramid of Giza (King Khufu’s pyramid). The isolate was studied and characterized by microscopic morphometric measurements, evaluation of enzymatic activities, and genotyping techniques. The isolate was identified as *Hortaea werneckii*, a pleomorphic black yeast that naturally inhabits hypersaline environments and infects human skin. It has been reported from humid temperate, subtropical, and tropical zones, mainly from marine habitats and adjacent areas, and is associated with marine life. Since it was observed in an unusual habitat, it raises the question of its type and origin, whether environmental or clinical. The Egyptian *Hortaea werneckii* GPS5 isolate was profiled and characterized by adaptive extremophilic tolerance to arid salt stress, low portability to infect human skin, and the capability of solubilizing calcite; besides it was phylogenetically clustered with previous recorded environmental accessions. A profile that matches the biodeterioration fungal agents known as rock-inhabiting fungi, a potential threat to cultural heritage sites that requires attention and prevention plans.

## Introduction

*Hortaea werneckii* is a halophilic black yeast of the family Teratosphaeriaceae, order Capnodiales. It is considered one of the most extensively studied black yeast and a model organism for halotolerant mechanism in Eukarya (Plemenitaš et al., 2014; Gunde-Cimerman et al., 2018). It is characterized by being halotolerant, producing melanin, and reproducing clonally *in vitro* a meristematic form with an unknown sexual state. Those characteristics are shared with phylogenetically related rock-inhabiting fungi and biodeterioration agents of the class Dothideomycetes (*Pseudotaeniolina globosa*; Rizk et al., 2021).

Among yeasts, *H. werneckii* is a pleomorphic black yeast that alternates between yeast and filamentous phases. It is an etiological agent that causes human skin mycosis known as “Tinea nigra” (Bonifaz et al., 2008). However, several non-clinical environmental isolates have been reported worldwide (Formoso et al., 2015). Its natural niches are hypersaline environments (up to 30% NaCl) and infected human skin (Butinar et al., 2005). The reports revealed that it occurs in diverse habitats from temperate to subtropical and tropical climatic zones, mostly seawater and adjacent eutrophic regions and coastal lines (e.g., beaches, deep-sea sediments, salterns, salt lakes, and salt marshes; Cantrell et al., 2006; Gunde-Cimerman et al., 2009). It was also associated with marine life (e.g., corals, dried and salted fish, microbial mats in salterns, sponges, xerophytes, and halophytes; Butinar et al., 2005; De Leo et al., 2019).

At the physiological level, *H. werneckii* tolerates high concentrations of salts and overcomes sodium toxicity typically known for hypersaline environments (Brauers et al., 2001). The expression of gene sets that produce compatible solutes and osmolytes modifies the cell membrane lipid composition of alkali-metal cations (Gostinèar et al., 2011). Within the species, a wide array of physiologically, morphologically, and phylogenetically diverse genotypes exhibit adaptive variation to produce assimilative, growing thallic hypersaline environments (Marchetta et al., 2018).

The combination of phenotypic arrays and enzymatic activity has contributed to the complete characterization of several strains from different habitats (Zalar et al., 2019). However, genotyping using ITS, TEF1, Beta-tubulin, LSU, and AFLP could not distinguish between pathogenic and non-pathogenic isolates even when the isolation source was used as *a priori* (Elsayed et al., 2016; De Leo et al., 2019; Zalar et al., 2019).

The mechanisms of biodeterioration have many common features, such as biofilm formation and discoloration, as well as the physical penetration by microorganisms (Sterflinger, 2010; Isola et al., 2016). Meanwhile, black fungi can lead to further encrustation and exfoliation after fungal hyphae into the stone matrix. The dark discoloration or blackening is often attributed to the formation of calcium sulfate or gypsum, which contributes to the formation of a black crust caused by the colonization of black fungi (Urzí and Realini, 1998; Dakal and Cameotra, 2012; Negi and Sarethy, 2019). Discoloration of stone monuments and buildings can cause mechanical damage and create an aesthetic appearance problem.

Biodeterioration of stone monuments is an extremely complex process involving biological, chemical, and environmental processes (Griffin et al., 1991). The present study aimed to isolate and investigate a microcolonial fungus observed on the interior walls of the royal corridor of king Khufu’s chamber in the Great Pyramid of Giza in Egypt. The isolate was identified and characterized at the morphological, physiological, and molecular genetic levels by microscopic morphometric measurements, assessment of exoenzymatic activities, and genotyping techniques, respectively.

## Materials and methods

### Sampling location

The Great Pyramid of Giza (Khufu’s Pyramid) is the oldest, largest, and most famous pyramid in the Giza pyramids complex, located in Memphis in the middle of the flood plain on the west side of the bank of the Nile River, Giza in Greater Cairo, Egypt, situated at 29°58′45 ′′N and 31°08′03 ′′E. It was completed around 2560 B.C. and originally stood 481 feet high (now 455 feet, since the outer shell and capstone have been removed). Khufu, also known in historiography by the Greek name “Cheops,” was the second pharaoh of the Fourth Dynasty of ancient Egypt during the Old Kingdom period (2589-2566 B.C.; Romer, 2007). The Great Pyramid of Khufu is the oldest of the “Seven Wonders of the Ancient World” and the only one still largely intact.

In the sampling area, the humidity ranges from 30% in May to 70% in November, with an annual average of 46%. According to bioclimatic variables of the WorldClim database, the minimum temperature in the coldest month is 6.5°C, while the maximum temperature of the warmest month is 34.8°C (Fick and Hijmans, 2017). The Giza pyramid complex is accessible to tourists and visitors, it received up to 13.1 million visitors *per* year in 2019 and dropped to 3.4 million in 2020 during the COVID-19 pandemic situation and raised back to 6.5 million visitors in 2021 (Reuters, 2020; News.CN, 2021).

### Fungal strain isolation

**FIGURE 1:**
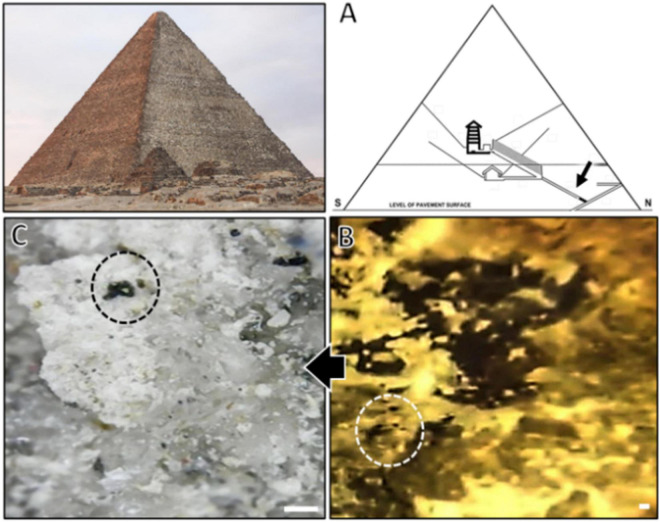
**(A)** Schematic drawing of Khufu’s Great Pyramid showing the sampling site in the pyramid; **(B)** a zoomed micrograph of the spotted site; **(C)** stereomicroscopic micrograph showing black spots presumably composed of black fungi growth (circles) visible over the sampled crust surface (scale bar is 1 mm).

Sample from the inky growth of the observed black spots was obtained from Khufu’s pyramid ascending passage that slopes to the grand gallery of the corridor ascending toward King Khufu’s chamber, an interior site with no or very low light at the corridor entrance (Figure 1). A crust was collected in a sterile bag for culture analysis on Dichloran Rose Bengal Chloramphenicol (DRBC) agar medium (#CM0727, Oxoid, USA) and incubated at 25°C for 2 weeks to 1 month. All individual morphotypes were transferred to Petri dishes containing malt extract agar (MEA, Oxoid, Basingstoke, United Kingdom) and incubated at 25°C to differentiate separate fungal colonies; the dominance of appearance among the morphotypes was with a black yeast morphotype, which was used for morphological and molecular analyses.

### Genotyping of *Hortaea werneckii* isolate

#### DNA extraction and PCR sequencing

Genomic DNA was extracted according to de Hoog and van den Ende (1992). The internal transcribed spacer (ITS) region and the small subunit of the nuclear ribosomal RNA 18S rRNA (SSU) were amplified with primers ITS1-ITS4 and NS1-NS24, respectively (White et al., 1990; O’Donnell, 2000). PCR reactions were performed using the MyTaq*™* Red Mix (Cat# BIO-25043, Bio-Line, UK). Each 25 μl reaction tube contained 5 pmol of each primer and 50 ng of template DNA. Amplification was performed using a Techne*™* 512 thermal cycler (Techne, UK). PCR programs were adjusted according to the primer pair melting temperature (Tm) as follows: The initial denaturation step at 95°C for 5 min was followed by 33 cycles of denaturation at 95°C for 30 s, annealing at 50°C (ITS) and 52°C (SSU) for 30 s, extension at 72°C for 30 s, and a final extension at 72°C for 5 min. PCR products were visualized by 1.5% agarose gel electrophoresis in 1x TBE buffer. All PCR reactions, when successful, were purified using the GeneJET*™* PCR Purification Kit (Fermentas, K0702) prior to automated Sanger sequencing (Macrogen, South Korea).

#### Phylogenetic analysis

Sequences were trimmed, assembled, and aligned using Geneious Prime (Kearse et al., 2012). BLAST searches in GenBank for species identification using NCBI online Blast tool.^1^ Retrieved sequences were revised and checked using the GenBank nucleotide database.^2^ Datasets included the newly generated sequences along with the NCBI-deposited sequences of *H. werneckii*. Phylogenetic inference was conducted using the maximum-likelihood method (ML), and clustering bootstrap support was confirmed by the Bayesian inference method (BI). The ML analyses were performed using FastTree V2 (Price et al., 2010) with 500 bootstrap replicates and default parameters. The BI analysis was performed by MrBayes 3.2 (Ronquist et al., 2012), where two parallel four chains were run for 1 million generations.

### Morphological characterization of *Hortaea werneckii*

The *H. werneckii* isolate GPS5 used in the present study was plated from a single colony on malt extract agar (MEA, Oxoid, Basingstoke, United Kingdom), potato dextrose agar (PDA; #1022, Condalab, Spain), and Oatmeal Agar (OMA; #2060, Condalab, Spain) and incubated at 25°C for up to 1 month. Colonies’ diameter, color, and structure were recorded after 2 weeks of incubation. The hexadecimal color-coding system (also known as Hex code) was used to describe the color of the colonies using the online tool https://htmlcolorcodes.com/color-picker/.

The microscopic study was performed using light phase contrast microscopy on a glass slide, using 2-week-old culture from the colony center and the colony margin mounted in 60% lactic acid for observation using Leica DMLB Tilting Trinocular Phase Contrast and Dark Field Light Microscope. Micrographs were captured using a Leica DFC500 digital color camera optimized with Micromax Arkon software (V8.12.05).

### Halotolerance and thermotolerance tests in solid media

The GPS5 isolate was cultured from a single colony on an MEA medium supplemented with various concentrations of NaCl (0, 10, 20, 25, and 30%; w/v) and incubated at 25°C for up to 2 months. The colony morphology and structure characterization, yeast (Y), filamentous (F), or yeast-filamentous (Y-F) were measured. The exact NaCl concentration of (0, 5, 10, 15, 20, 25, and 30%; w/v) was used to evaluate thermotolerance at different temperatures (0, 10, 20, 25, and 37°C) during 2-month incubation. The average growth of the experiment was evaluated as good, weak, or no growth following Zalar et al. (2019).

### Exozyme activity

Enzymatic activity assay was performed using fresh cultures of GPS5 isolate and inoculated on specific culture media for each enzyme activity; all assays were performed in triplicate.

#### Plant degrading enzymes

Amylase activity was tested using media containing 0.2% soluble starch, 0.67% yeast nitrogen base (YNB), and 2% agar. After incubation at 25°C/1 week, an iodine solution was added to the plates; the positive result appeared as a clear halo around the enzyme-producing colonies (Hankin and Anagnostakis, 1975).

The activity of lipids or long-chain esters (i.e., lipolytic and esterase) was detected on two different media, according to Brizzio et al. (2007). The first was the Tween 80 agar medium (Slifkin, 2000) composed of 2.5% Tween 80, 1% peptone, 0.5% NaCl, 0.01% CaCl_2_, 0.1% glucose, and 2% agar without and with the addition of 5 and 10% NaCl (pH was adjusted to 6.8). The second was a tributyrin agar medium consisting of 1% tributyrin, 0.5% peptone, 0.3% yeast extract, and 2% agar (Sierra, 1957). After incubation at 25°C/1 to 2 weeks, a white halo around the colonies indicated positive enzymatic activity.

The β-glucosidase activity was tested on a medium containing 0.67% YNB, 1% esculin, 2.0% agar, and 1% ammonium ferric citrate solution added to the medium with a final concentration of 0.02% the positive β-glucosidase activity, visualized as dark brown coloration around the colony producing enzyme (Strauss et al., 2001).

#### Animal-related enzymes

Phospholipase activity was tested according to Price et al. (1982). The medium consisted of 1% peptone, 2% glucose, 5.73% sodium chloride, 0.05% calcium chloride, and 2% agar. The sterile egg yolk was added at a final concentration of 4% when the medium had cooled down to 50°C after autoclaving, then incubated at 30°C for 14 days. A clear zone around the colonies is observed when the enzyme activity is positive.

Urease activity was measured in yeast nitrogen base (YCB) urea agar tubes (1.17% YCB, 0.02% acid fuchsin, 2.0% agar, and 2.0% urea) and incubated at 37°C for 72 h according to Maldonade et al. (2007). When the color of the medium turns white, this indicates positive urease activity.

Protease activity was tested with casein and gelatin substrates. The assay for casein was performed on media consisting of 0.5% casein, 0.5% glucose, 0.67% YNB, and 2% agar, pH 7.0, and without and with the addition of 5% and 10% NaCl. After incubation at 25°C for 7 days, casein digestion was seen as a degradation halo around the colonies after adding 1N HCl for 1 h (Montville, 1983). The assay for gelatin was performed according to the method of Smith and Goodner (1958). Using a medium containing 1.2% gelatin, 0.4% peptone, 0.1% yeast extract, and 1.5% agar without and with the addition of 5 and 10% NaCl. The degradation halos around the colonies were observed after adding 4.1M (NH_4_)_2_SO_4_.

Elastin digestion was tested on 1 g of elastin powder (Worthington) added to 100 ml of Trypticase Soy Agar (TSA; Oxoid) at pH 8.0 and incubated at 25°C for 2 weeks. The result was seen as a degradation halo around the colonies. If the clear zone did not appear, an aqueous solution of saturated ammonium sulfate was added to the plates to enhance the clear zone (Muhsin et al., 1997).

DNase activity was measured using a similar procedure for detecting DNase production by staphylococci (Sinicropi et al., 1994). On DNase agar complexed with methyl green (Oxoid, U. S. A.), a clear zone around the colonies indicated DNase activity after incubation at 25°C and 37°C for up to 2 weeks.

### Calcium carbonate solubilization test

The potential ability of *H. werneckii* isolate GPS5 to dissolve calcite was demonstrated on CaCO_3_-glucose agar medium (glucose 1%, CaCO3 0.5%, agar 1.5%; pH adjusted to 8.0). The test was performed on 1 cm^2^ agar blocks and incubated at 30°C for 8 weeks (Santo et al., 2021).

## Results

### Morphological characterization of *Hortaea werneckii*

**FIGURE 2:**
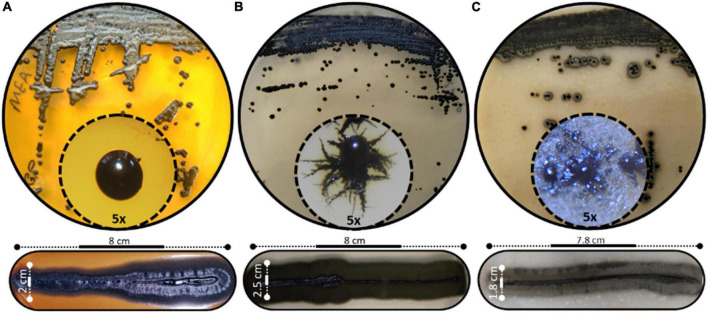
Different scopes of *Hortaea werneckii* isolate GPS5. The progressive morphological growth as a colony with a closer 5_x_ zoom using a stereomicroscope is shown (above), and the line strike over three tested culture media (below). **(A)** MEA, **(B)** PDA, and **(C)** OMA.

The morphological characteristics of *H. werneckii* GPS5 isolate on the three tested culture media MEA, PDA, and OMA were initially started as a small yeast-like. Shiny circular colony with convex elevation and entire margin with pearl black color (Hex code #060809) of 2.0–8.0 mm on MEA. Additionally, on PDA, the growth was dark olive green (Hex code #111509) yeast-like with a rhizoid-like structure at the colony margin and reached a 2.5–9.0 mm diameter. For OMA, a yeast-like growth surrounded by a superficial gray-olive aerial filamentous margin (Hex code #403e33) reaches 3.0–10 mm diameters after 14 days (Figure 2).

**FIGURE 3:**
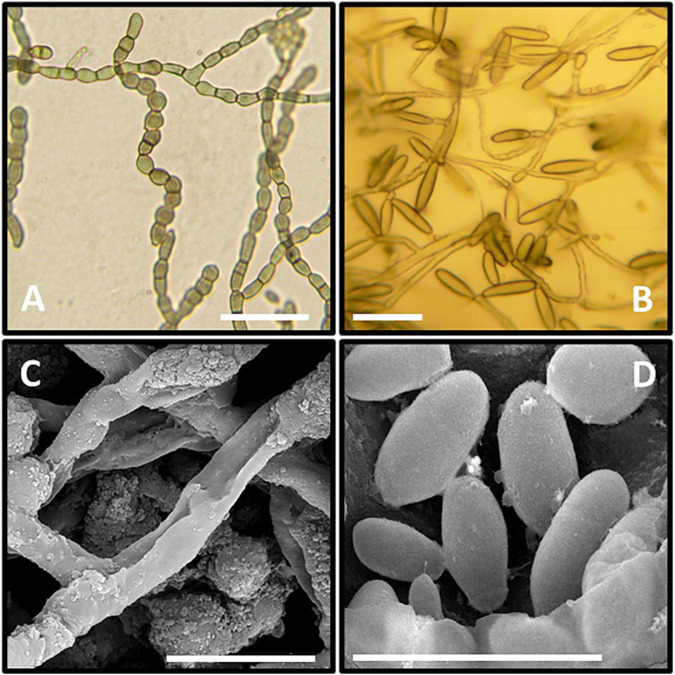
Microscopic observation of GPS5. **(A–C)** Clumps of meristematic hyphae; **(C,D)** mature conidia. **(A–C)** Micrographs under the light microscope. **(C,D)** Micrographs under the scanning electron microscope. Bar = 1 cm, black bars = 20 μm.

The microscopic observations of the size of the hyphal and conidial structures were recorded on MEA media. The aerial immersed mycelia consisted of a hyphal cell that measured 4.6 μm diameter length and 3.2 μm in width, with 2.5 μm septa. All observed conidia were one-celled types and measured average diameter lengths of 6.1 μm and 2.6 μm width (Figure 3).

### Halotolerance and thermotolerance in solid media

**FIGURE 4:**
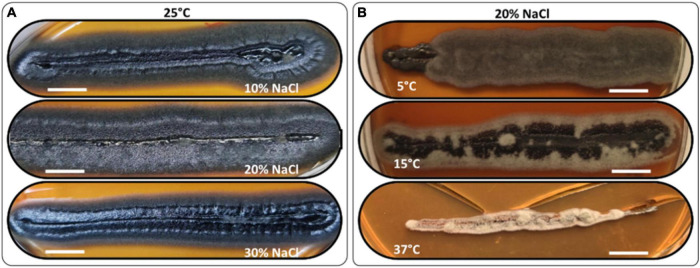
Morphology examples of *H. werneckii* isolate GPS5 halotolerance **(A)** and thermotolerance **(B)** line-streak growth over MEA media supplemented with 10, 20, and 30% NaCl at 25°C for 2 months and thermotolerance growth on MEA media supplemented with 20% NaCl at 5°C, 15°C, and 37°C, over 2 months. Bar = 1 cm.

The *H. werneckii* isolate GPS5 halotolerance ability was evaluated under different salinity concentrations at 25°C. A scale of 0, 5, 10, 20, 25, and 30% NaCl (w/v) was added to MEA media considering colony diameter and line-streak growth. The results for the colony diameter in mm showed that the strain could grow without and with all NaCl concentrations. The colony diameter at both 0 and 5% NaCl was 8.5 mm, and at 10 and 15% was 9.4 mm. The isolate achieved optimal growth with a colony diameter of 13.8 mm at 20% NaCl, while increasing the NaCl concentration to 25 and 30%, the colony diameter was reduced to 7.6 and 5.2 mm, respectively. Line-streak growth was recorded as strong at all concentrations (Supplementary Table 1). The growth aspect at the beginning was pearl black (Hex code #141c20) yeast on the center with a filamentous form at the colony margin and turn to black-gray color by aging (Hex code #262a31; Figure 4A).

The thermotolerance test was performed as a line-streak growth on MEA media supplemented with a scale of 0, 5, 10, 15, 20, 25, and 30% (w/v) NaCl concentrations and incubated at 5, 15, 25, and 37°C. Growth over 2 months was recorded as good, weak, or no growth (Supplementary Table 2). At 25°C, the growth was strong at all NaCl tested concentrations. At 5°C, the growth was strong for 0 and 20% and grew weaker for 10 and 30% NaCl, all grew filamentous forms with charcoal color (Hex code #494645). At 10°C, the growth was strong for 0 and 10% and weaker at 20 and 30% NaCl, all grew filamentous forms with hard-coal gray color (Hex code #636262). At 15°C, the growth was strong from 0 to 20% and was only weak at 30% NaCl, the growth was filamentous black smoke (Hex code #3f3f41) in the center and cold gray on the edge (Hex code #9f9f9f). At 37°C, the growth was exclusive to 10 and 20% NaCl with filamentous free melanin growth of chert color (Hex code #868082; Figure 4B).

### Enzymatic activities and calcium carbonate solubilization ability

**FIGURE 5:**
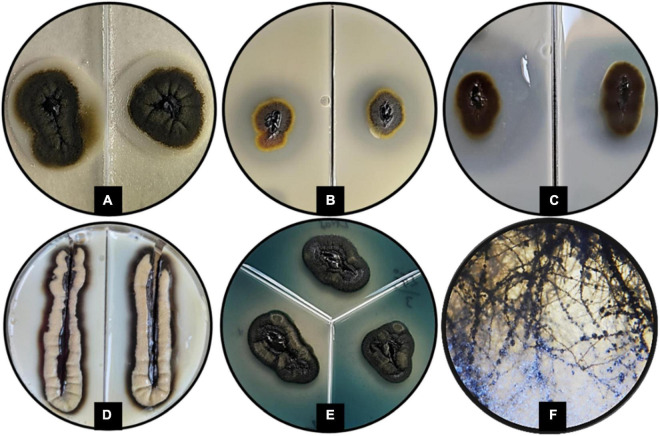
Enzymatic activity examples of *H. werneckii* GPS5 isolate. **(A)** Esterase activity on 10% NaCl at 37°C; **(B,C)** proteolytic activities on casein and gelatin 5% NaCl at 25°C, respectively; **(D)** phospholipase activity at 25°C; **(E)** DNase activity at 25°C; **(F)** colony growth in CaCO_3_ agar.

The GPS5 isolate was able to express various enzymes. The extracellular enzyme activity generally was absent at 0% NaCl, weak at 10%, and strong at 20% of NaCl. The proteolytic activity on casein was strong at 0 and 5% NaCl for both 25°C and 37°C, weak for 10% NaCl at 25°C, and absent at 37°C. In comparison, proteolytic activity on gelatin was negative at 0% NaCl for both 25°C and 37°C, while it was strong at 5 and 10% NaCl at 25°C and recorded weak activity at 37°C. The esterase activity without NaCl was weak at both 25°C and 37°C, and it became stronger with 5% NaCl at 25°C and 37°C, while at 10% NaCl, the growth was strong at 37°C and weak at 25°C (Supplementary Table 3). The amylase and phospholipase activities were strong at 25°C. For DNase and urease, the activity was strong at 25°C and weak at 37°C. The activity was absent for B-glucosidase and lipase at 25°C, and for elastin hydrolysis at 30°C (Figures 5A–E and Supplementary Table 4). The potential ability of *H. werneckii* strain GPS5 to solubilize calcite was tested on a CaCO_3_ medium. It was found positive as a clear zone of calcium carbonate precipitation was seen around the colony growth (Figure 5F).

### Molecular characterization and genotyping

**FIGURE 6:**
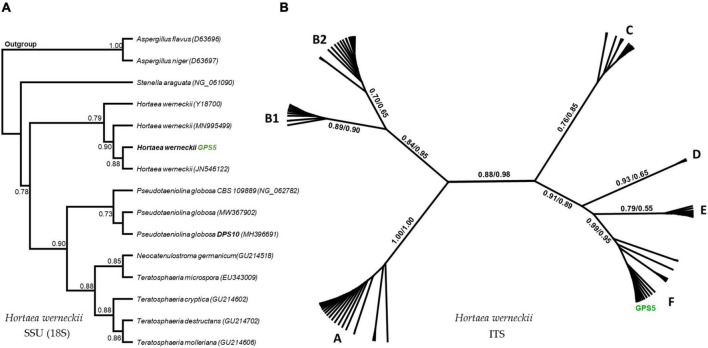
Maximum-likelihood phylogenetic trees based on SSU and ITS regions. The rooted SSU tree shows the GPS5 isolate within the *H. werneckii* clade **(A)**. The ITS tree includes all data recorded for *H. werneckii* in the NCBI database, where different clusters were named by letters, while ML/BI bootstrap supports are written in numbers near each node **(B)**.

The SSU-based tree computed by the maximum-likelihood method was rooted by the other Capnodiales species in the tree, resulting in a highly supported clade that includes GPS5 with other *H. werneckii* accession from GenBank (bootstrap support value = 0.97). The clade was proximate to *Stenella araguata* and a clade of *P. globosa* isolates, that included an isolated specimen from a similar sampling area (i.e., Djoser pyramid; DPS10; Figure 6A).

The ITS-based tree computed by the maximum-likelihood method was visualized in an unrooted form, including all the available *H. werneckii* accessions (100 accessions including uncultured fungi) and the GPS5 isolate. The tree was alphabetically divided into seven distinguished clusters; each cluster was monophyletic except for cluster B, which was created by two subclusters.

The Egyptian isolate was highly supported within the cluster F (bootstrap value = 0.99 ML and 0.95 BI), a cluster that included genotypes mostly isolated from hypersaline sources, e.g., continental shelf sediments (KM014604; KM014589), endophytic fungi of the coastal plant *Ipomea pes-caprae* (MK336620), methane hydrate-bearing deep-sea marine sediments (DQ300280), associated with a marine sponge (*Haliclona simulans* and *Gelliodes carnosa*; FJ755827), in addition to one non-human clinical strain isolated from superficial infectious lesions of a guinea pig in Japan (AB087199) and two isolates of unstated sources from Brazil and Mexico (CBS 708.76 and CBS 126986; Figure 6B).

## Discussion

Isolation of black yeasts is challenging due to their peculiar nutritional nature and the inability to prohibit similar halotolerant acidophilic fungi that grow at higher rates than the black yeasts under the same isolation conditions (e.g., high salt concentration and temperature around 20 ± 5°C; Sterflinger, 2006). The *H. werneckii* isolate GPS5 was able to grow at all NaCl concentrations (0–30%), while the optimum growth was at 20% at 25°C with pearl black shiny yeast center and filamentous black-gray at margins, a common aspect for several genotypes of *H. werneckii* (Zalar et al., 2019), the identification was completed using SSU and ITS sequencing and phylogenetic analysis.

In the *H. werneckii* case, the temperature is essential in studying and describing its isolates. For example, the yeast phase can be observed at low temperatures (15 ± 5°C), while the hyphal phase can be observed at higher temperatures (30 ± 5°C; Elsayed et al., 2016). However, the morphological aspect of the GPS5 growth at different temperatures mainly was filamentous, while the yeast phase was absent except for its growing without NaCl at 25°C on MEA and PDA media. Salt type and concentration might affect hyphal growth and shape; e.g., zigzag hypha was noticed in *Ashbya gossypii* and *Histoplasma capsulatum* when grown in a media with high calcium content (Tian and Shearer, 2002; Bauer et al., 2004). Additionally, the repetitive culturing of *H. werneckii* was found to force phenotypic variations (Elsayed et al., 2016). The morphological plasticity/versatility of *H. werneckii* can be considered the primary challenge when describing *H. werneckii* genotypes (Zalar et al., 2019). Thus, careful inspection and measurement recording under the same laboratory conditions is an essential precaution for morphological assessment in *H. werneckii* studies.

*Hortaea werneckii* was commonly isolated from soil, humus, marine product (i.e., salted dried fish), and human skin in humid areas near coastal lines in tropical and subtropical regions (Prieto-Granada et al., 2010). Isolation sources with less humidity were reported, such as house dust, wood, and compost (Prieto-Granada et al., 2010). The *H. werneckii* previously reported from Egypt was presented by three pleomorphic ecotypes isolated from salt marches in the Northern parts of Egypt (Elsayed et al., 2016). The three were phylogenetically clustered in two separate branches, and none was clustered with the GPS5 isolate. Salt marshes and wetlands are flooded and drained by salts, thus, the presence of *H. werneckii* inhabiting a desiccated stone surface in a hyper-arid condition is remarkable. A case was never reported from Egypt and rarely reported worldwide (Prieto-Granada et al., 2010; Elsayed et al., 2016). The optimum growth of the GPS5 strain was at 25°C on 20% NaCl, higher than the range previously reported (i.e., 25°C at 10% NaCl; Elsayed et al., 2016; Zalar et al., 2019). Exceptionally, the GPS5 grew normally on MEA media supplemented with 30% NaCl at 25°C, a salinity level that shows low occurrences (CFU^–1^) even for halophilic melanized fungi isolated from solar salterns (Butinar et al., 2005). Salt-related adaptability mechanism would explain the high halophilic and halotolerant ability of the GPS5 compared to other wetlands *H. werneckii* isolates (Elsayed et al., 2016; Zalar et al., 2019). A remarkable level of tolerance requires additional genomic and transcriptomic sequencing to define its complexity and nature.

Since the great pyramid of Giza is a unique archeological site that was and still is a place of attraction for nationals and tourists to visit through the ages, it was important to detect the pathogenicity potential of the GPS5 isolate and to understand its origin and type, whether it was an environmental or clinical source existed through human visits to the pyramid interiors. The GPS5 isolate was halophilic and halotolerant, a general ability that characterizes the species regardless of the sampling source or habitat (Zalar et al., 2019). The susceptibility of *H. werneckii* to several antifungal agents (e.g., itraconazole (Gupta et al., 1997; Ng et al., 2005), ketoconazole (Ng et al., 2005; Ortega-Morales et al., 2016), and voriconazole; Ng et al., 2005) was previously reported to affect clinical as well as environmental isolates (e.g., ecological isolates from Brazil; Ortega-Morales et al., 2016); thus, it was not included in our analysis to define the GPS5 pathogenicity to humans. Therefore, the enzymatic activities were assessed to interpret the pathogenic nature of clinical and environmental *H. werneckii* isolates (Formoso et al., 2015; Zalar et al., 2019). At 37°C (i.e., mimicking human body temperature), the GPS5 isolate lost its ability to grow on MEA media without or with 30% NaCl, and the overall enzymatic activity was weak or absent. The enzymatic activity profile of the GPS5 isolate was dominated by animal-related enzymes, like phospholipase, caseinase, and gelatinase and few plant degrading enzymes (i.e., amylase, and esterase). Our isolate profile contradicts the profiles from previous reports of *H. werneckii* isolates (e.g., Zalar et al., 2019), where the major profile was dominated by plant degrading enzymes. Even though the GPS5 isolate produced DNase at lower levels at 37°C, it was unable to digest the urea efficiently nor produce elastase, an essential enzyme to infect human skin in dermatophytes (Muhsin et al., 1997). Moreover, the GPS5 isolate was phylogenetically clustered with other isolates mostly found in environmental biological samples or associated with hypersaline habitats.

Black fungi are considered major agents of microbial deterioration of building stones. Their occurrences were associated with well-documented effects, such as aesthetic damage due to stone darkening and other color changes, besides surface erosion and flaking (Schiavon et al., 2013; Caneva et al., 2015; Isola et al., 2016; Ortega-Morales et al., 2016). For example, the Angkor sandstone monuments showed a color change from white to dark green and black (Hu et al., 2013), marble and limestone blackening in darkened areas of the exterior parts of the Florence Cathedral of Italy (Santo et al., 2021), black crusts with distinctive biodeterioration patterns of D. Afonso I (First Portuguese King) tomb in Portugal (Trovão et al., 2020), ancient Roman stone artworks at Vatican City and historical buildings in Cagliari of Italy (Isola et al., 2016), the Mayan temple of Kukulkan Mexico (Ortega-Morales et al., 2016), and Rock-Hewn Churches of Ethiopia (Schiavon et al., 2013). The ability of the isolated fungi from the mentioned examples to dissolve calcium carbonate has been reported (e.g., genera Aspergillus, Aureobasidium, and Cladosporium; Trovão et al., 2020; Santo et al., 2021) that confirmed their ability to damage and affect stone structures. Fungus-assisted degradation of limestone initiated with the secretion of organic acids to dissolve the stone substrate and increase the alkalinity of the microhabitat, then produce extracellular polymeric substances to chelate-free calcium ions in a process known as biomineralization (e.g., fungus *Colletotrichum acutatum*; Li et al., 2018). In some reports, the biomineralization of cultural heritage sites by fungal biofilms was reported to contribute to the rock coating development (Gadd and Dyer, 2017). However, the GPS5 isolate was observed in a microcolonial form with the ability to dissolve calcite and form calcium carbonate precipitations, thus highlighting it as a potential biodegrade for the inhabited limestone surface. Observations suggest that the GPS5 is an inland, environmental, biodegradation agent and a stone-inhabiting genotype of *H. werneckii*, and the Great Pyramid of Giza is its natural habitat.

## Data availability statement

The datasets presented in this study can be found in online repositories. The names of the repository/repositories and accession number(s) can be found below: https://www.ncbi.nlm.nih.gov/genbank/ (OP009760 and OP009761).

## Author contributions

SR and MM: conceptualization, investigation, visualization, and project administration. SR: methodology, resources, data curation, writing – original draft preparation, and formal analysis. MM: software, validation, writing – review and editing, supervision, and funding acquisition. Both authors have read and agreed to the published version of the manuscript.

## Footnotes

1
https://blast.ncbi.nlm.nih.gov/Blast.cgi

2
https://www.ncbi.nlm.nih.gov/nuccore
